# A maximum likelihood QTL analysis reveals common genome regions controlling resistance to *Salmonella* colonization and carrier-state

**DOI:** 10.1186/1471-2164-13-198

**Published:** 2012-05-21

**Authors:** Tran Thanh-Son, Beaumont Catherine, Salmon Nigel, Fife Mark, Kaiser Pete, Le Bihan-Duval Elisabeth, Vignal Alain, Velge Philippe, Calenge Fanny

**Affiliations:** 1 INRA, UR83 Recherches Avicoles, F-37083, Nouzilly, France; 2 Institute for Animal Health, Compton Berkshire, RG20 7NN, UK; 3 The Roslin Institute & R(D)SVS, University of Edinburgh, Easter Bush, Midlothian, EH25 9RG, UK; 4 INRA UR 0444 Laboratoire de Génétique Cellulaire, F-31326, Auzeville, France; 5 INRA, UR 1282 IASP Infectiologie Animale et Santé Publique, F-37083, Nouzilly, France

## Abstract

**Background:**

The serovars Enteritidis and Typhimurium of the Gram-negative bacterium *Salmonella enterica* are significant causes of human food poisoning. Fowl carrying these bacteria often show no clinical disease, with detection only established post-mortem. Increased resistance to the carrier state in commercial poultry could be a way to improve food safety by reducing the spread of these bacteria in poultry flocks. Previous studies identified QTLs for both resistance to carrier state and resistance to *Salmonella* colonization in the same White Leghorn inbred lines. Until now, none of the QTLs identified was common to the two types of resistance. All these analyses were performed using the F2 inbred or backcross option of the QTLExpress software based on linear regression. In the present study, QTL analysis was achieved using Maximum Likelihood with QTLMap software, in order to test the effect of the QTL analysis method on QTL detection. We analyzed the same phenotypic and genotypic data as those used in previous studies, which were collected on 378 animals genotyped with 480 genome-wide SNP markers. To enrich these data, we added eleven SNP markers located within QTLs controlling resistance to colonization and we looked for potential candidate genes co-localizing with QTLs.

**Results:**

In our case the QTL analysis method had an important impact on QTL detection. We were able to identify new genomic regions controlling resistance to carrier-state, in particular by testing the existence of two segregating QTLs. But some of the previously identified QTLs were not confirmed. Interestingly, two QTLs were detected on chromosomes 2 and 3, close to the locations of the major QTLs controlling resistance to colonization and to candidate genes involved in the immune response identified in other, independent studies.

**Conclusions:**

Due to the lack of stability of the QTLs detected, we suggest that interesting regions for further studies are those that were identified in several independent studies, which is the case of the QTL regions on chromosomes 2 and 3, involved in resistance to both *Salmonella* colonization and carrier state. These observations provide evidence of common genes controlling *S.* Typhimurium colonization and *S*. Enteritidis carrier-state in chickens.

## Background

Bacteria belonging to the *Salmonella enterica* species are responsible for diseases in several animal species and in humans. *Salmonella enterica* serotypes Enteritidis and Typhimurium are two of the main sources of human food poisoning. One of the most important factors is that poultry can carry these bacteria without any clinical symptoms, and thus the carriage of disease goes unnoticed prior to culling. Selection and breeding of chickens more resistant to *Salmonella* carrier state, i.e. able to rapidly clear *Salmonella*, could reduce the spread of these bacteria in poultry stocks and hence improve food safety. Selection for resistance to carrier-state requires the use of genetic markers in poultry breeding programs. Identification of such markers by several authors has been done using different approaches. These include candidate gene analyses, genome scans with single marker analyses, quantitative trait loci (QTLs) detection studies by interval mapping with microsatellites and more recently SNP markers (reviewed in [1]).

Several QTLs involved in the genetic control of resistance to *Salmonella* carrier state have been identified in experimental inbred lines derived from White Leghorn laying hens. A first study was undertaken using a selective genotyping approach (i.e. animals with extreme phenotypes were chosen) with only partial genome coverage [2]. Several QTLs for resistance to carrier-state were identified in two crosses between the experimental inbred lines N and 6_1_: one genome-wide significant QTL on chromosome 2 and five chromosome-wide significant QTLs on chromosomes 1 (2 QTLs), 5, 11 and 16. Two of these QTLs, on chromosomes 2 and 16, were confirmed in a subsequent analysis including all animals, while those on chromosomes 1 and 16 were replicated in an independent population of a commercial line of laying hens [3]. More recently a new, more powerful QTL analysis was carried out [4], using a higher number of animals genotyped with a higher number of markers (480 SNPs). Using this approach, we were able to perform a more extended genome scan which resulted in the identification of novel QTLs on several microchromosomes, not previously covered in other analyses. Among them, one QTL on microchromosome 14 was significant at the genome-wide level. However, although half of the phenotypic data used were the same as in previous analyses, several of the QTLs previously observed were not detected in the new analysis, including the genome-wide significant QTL on chromosome 2 [2,3].

Similar (and in some cases the same) White Leghorn inbred lines have also been used to identify genome regions controlling resistance to acute salmonellosis. The major QTL *SAL1* was originally identified in a backcross between lines 6_1_ and 15I on chromosome 5 [5]. Its location has recently been refined in a further analysis [6]. In addition, four QTLs controlling resistance to *Salmonella* colonization have been detected in a (6_1_xN)xN backcross on chromosomes 2, 3, 12 and 25 [7]. Until now, none of the QTLs controlling carrier-state has been identified close to those QTLs for resistance to colonization or systemic salmonellosis. This is why it was hypothesized that the genetic controls of *Salmonella* carriage and colonization were independent [2]‐[4]. Nevertheless, in our last genome scan performed with QTLExpress, an effect nearing significance could be observed on chromosome 3. In addition, our earliest QTL analyses identified a QTL on chromosome 2 [2,3], although it was not confirmed in our last SNP genome scan [4].

These apparently inconsistent results may be partly dependent on the method of QTL analysis. All were performed using the F2 inbred option of the QTLExpress software [8]. This option relies on the hypothesis that all F1 fathers are heterozygous and share a common QTL effect, which is only partially true in this case. The aim of the present study was therefore to perform QTL analysis using Maximum Likelihood with the QTLMap software, which does not require any assumption about fixation of the QTL alleles in the founder lines. With this software it was also possible to evaluate different QTL effects between sire families, to test the co-existence of two QTLs and to analyze discrete data. For these analyses we used the same data as previously described [4], i.e. 480 SNP genotypes for 378 animals already phenotyped. This dataset was enriched with eleven SNP markers flanking the QTLs controlling *Salmonella* colonization identified by Fife et al. [7] on chromosomes 2, 3, 12 and 25 [7]. We were thus able to observe the impact on QTL detection of two different statistical approaches using the same set of data, whilst interrogating any overlap in the carrier state/colonization QTLs using increased marker density in the QTL regions controlling *Salmonella* colonization.

## Methods

### Animals

Briefly, as described previously [4], two progenies were considered. Both were F2 crosses between the experimental inbred White Leghorn lines N and 6_1_, each from different parents. The first one (called P1) comprised 185 F2 animals reared and phenotyped in 2005 and the second (P2) 193 F2 animals reared and phenotyped in 2007. Animals were reared at the PEAT unit (Pôle d’Expérimentation Avicole de Tours).

### SNP genotyping and mapping

As described previously [4], a set of 480 fully informative SNP markers was used to genotype the F2 animals and their parents and a genetic map was produced using the Cri-Map program. In addition, animals belonging to P2 were typed for 11 SNP markers flanking the four QTLs identified on chromosomes 2, 3, 12 and 25 in a (6_1_xN)xN backcross progeny [7]. The conformity of marker segregation with Mendelian inheritance rules was checked by Chi-square tests. New genetic maps of chromosomes 2, 3, 12 and 25 were built with Cri-Map using only the genotypes obtained from P2.

### *Salmonella* challenges

As previously described, experimental infections of both F2 progeny were performed at the PFIE (Plateforme d’Infectiologie Expérimentale) [9]. One-week-old birds were orally inoculated with 5x10^4^ bacteria from the *S*. Enteritidis phage type 4 (PT 4) strain 1009, which is a spontaneous nalidixic acid (NA) and streptomycin (SM) resistant mutant strain. Within each experiment all chicks were hatched on the same day. In P1, two cloacal swabs were taken 4 and 5 weeks p.i. and results expressed as log_10_ colony-forming units (cfu) and thereafter called CSW4 (cloacal swab week 4) and CSW5. Results were also expressed as a discrete trait for CSW4: 0 when no bacteria were found and 1 for positive results for the CSW4 trait (CSW4d). For both P1 and P2, chicks were sacrificed 5 weeks p.i. and the number of *Salmonella* cfu counted in the caeca. Caecal bacterial counts were expressed as log (cfu) per gram of caeca (CAEC). In brief, traits measured in P1 were CAEC, CSW4 and CSW5 while in P2 only CAEC was measured.

### QTL analysis software and significance thresholds

QTLs were identified in P1 and P2 separately using the QTLMAP software [10,11], which performs interval mapping based on maximum likelihood calculations [12]. Initially, single-QTL analysis was performed using the ratio of likelihoods under the hypothesis of one (H1) vs no QTL (H0). When no QTL was detected, this was followed by analysis for complex QTLs by comparing maximum likelihood under the hypothesis of two QTLs segregating (H2) versus that of the absence of QTL (H0). The model used for all analyses took into account the population mean. Likelihood was completely linearized as described in [10]. For the analysis of CSW4d, the model took into account the discrete distribution of the trait.

For each trait on each chromosome, the significance threshold at the chromosome-wide level was calculated from the results of 1,000 permutations performed under the null hypothesis of no QTL segregating. The genome-wide probability was further derived from the chromosome-wide probability using an approximate Bonferroni correction:

(1) $$\text{Pgenome}-\text{wide}=1\;-{\left(1\;-\text{ Pchromosome}-\text{wide}\right)}^{1/\text{r}}$$

in which r was obtained by dividing the length of a specific chromosome by the length of the genome considered for QTL detection (3,3918 cM), as in [2]. Confidence intervals for QTLs (95 %) were calculated by using the one-LOD drop-off method following Lander and Botstein [12].

### Candidate gene identification

For all QTLs detected using the one-QTL analysis, candidate genes were identified by the software AnnotQTL [13], available at http://annotqtl.genouest.org. This tool was designed to assist the characterization of genes in a QTL region as a step towards selecting the best candidate genes. It localizes the genes to a specific region using NCBI and Ensembl data and adds the functional annotations available from other databases (Gene Ontology, Mammalian Phenotype, HGNC and Pubmed). To limit the number of genes considered, for each QTL the interval considered was defined by the two SNP markers flanking the QTL peak.

## Results

### Additional SNP genotyping and mapping

The list of additional SNP markers and quality data describing them are detailed in Table 1. Marker IAH-12D was non-informative in our progeny. Marker IAH-2 C on chromosome 2 was in moderate distortion (p < 0.05) while markers IAH-3A, IAH-3B and IAH-12D were in very strong segregation distortion (p < 0.001). These markers must be considered with caution. New, enriched maps of chromosomes 2, 3, 12 and 25 were constructed based on the SNP genotypes collected from P2, excluding IAH-12D.

**Table 1:** **Quality data about the SNP markers flanking QTL for *Salmonella* colonization**[7]**added to the original SNP data set**[4]

**Marker**	**Chr**^**1**^	**Position (bp)**	**N**^**2**^	**Inf**^**3**^	**SD**^**4**^	**p**_**SD**_^**5**^
IAH-2B	2	19,869,778	182	23/23	NS	0.052
IAH-2 C		22,726,015	182	23/23	S*	0.010
IAH-2D		24,308,758	179	22/23	NS	0.130
IAH-3A	3	92,315,616	152	20/23	S***	0.000
IAH-3B		95,968,986	182	23/23	S***	0.001
IAH-3 C		110,245,601	184	23/23	NS	0.378
IAH-12B	12	14,781,290	181	23/23	NS	0.597
IAH-12 C		17,496,361	181	23/23	NS	0.704
IAH-12D		18,927,306	162	1/23	S***	0.000
IAH-25A	25	875,498	92	17/23	NS	0.093
IAH-25B		1,843,484	184	23/23	NS	0.556

^1^Chromosome.

^2^Number of genotypes available in the F2 Nx6_1_ progeny P2.

^3^Informativity: number of heterozygous F1 parents on the total number of F1 parents.

^4^Significance level of the segregation distorsion evaluated with a Chi-square test: NS non-significant; * significant with p < 0.05 ; *** significant with p < 0.001.

^5^Probabilities associated with a Chi-square test between genotypic frequencies expected according to Mendelian rules and observed frequencies.

### QTL analyses

**Figure 1:**
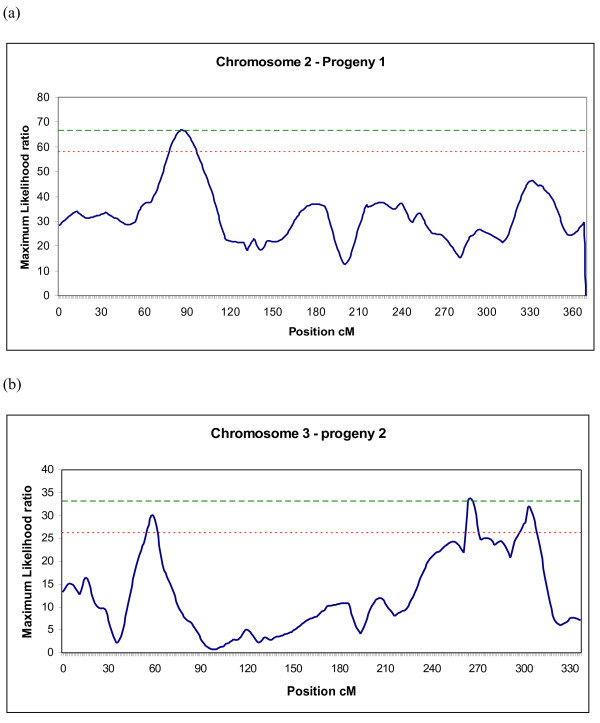
**Maximum likelihood ratio for trait CAEC on chromosome 2, progeny P1 (a) and chromosome 3 enriched with three markers close to a QTL for resistance to *Salmonella* colonisation, progeny P2 (b).** Results obtained from QTLMap software. **− − −**, 5 % genome-wide significance; - - -, 5 % chromosome-wide significant.

QTLs identified in this study are detailed in Table 2 and Figure 1 for one-QTL analyses and in Table 3 for two-QTL analyses. One-QTL analyses led to the identification of five QTLs in P1, on four chromosomes. One QTL was significant at the genome-wide level on chromosome 2 (for CAEC). The four other QTLs were significant at the chromosome-wide level and mapped on chromosome 19 (CAEC), 21 (CSW4), and 23 (CAEC, CSW4_d_). In P2, two QTLs significant at the chromosome-wide level were identified on chromosomes 3 and 26 for CAEC. The former was significant at p < 0.01 at the chromosome-wide level. Interestingly, with the enriched map of chromosome 3 the significance of this QTL was higher (p = 0.005). Using the other enriched maps did not lead to the detection of QTL on chromosomes 2, 12 and 25.

**Table 2:** Parameters associated with the QTLs identified in progeny 1 (P1) and progeny (P2) using a one-QTL test

**Chr**^**1**^	**Progeny**	**Trait**	**QTL position** **(cM)**	**C.I.**^**2**^ **(cM)**	**LRT**^**3**^	**Flanking markers**^**4**^**(Mb)**	***P*-value**^**5**^ **(Chr)**	**S.L.**^**6**^
2	P1	CAEC	86	80-92	66.83	31.8-38.3	<0.005	***
3	P2	CAEC	236	234-241	30.29	94.25-98.25	<0.01	**
19	P1	CAEC	21	17-29	56.01	2.71-3.80	<0.01	**
21	P1	CSW4	3	0-6	21.48	0.40-1.22	<0.05	*
23	P1	CAEC	10	7-15	50.25	1.32-2.74	<0.005	*
23	P1	CSW4_d_	1	0-6	46.83	0.72-1.32	<0.05	*
26	P2	CAEC	29	21-37	49.30	2.60-3.31	<0.05	*

^1^Chromosome number.

^2^1-LOD-drop confidence interval (cM).

^3^Likelihood Ratio Test.

^4^Positions of the two markers flanking the QTL peak.

^5^*P*-values obtained by 1000 chromosome-wide permutations.

^6^Significance level: *significant at P < 0.05 at the chromosome-wide level, ** significant at P < 0.01 at the chromosome-wide level, *** significant at P < 0.05 at the genome-wide level.

**Table 3:** Parameters associated with the QTLs identified in progeny 1 (P1) with a two-QTL test

**Chr**^**1**^	**Progeny**	**Trait**	**First QTL (cM)**^**2**^	**Second QTL ** **(cM)**^**3**^	**LRT**^**4**^	**Flanking markers Q1**^**5**^ **(Mb)**	**Flanking markers Q2**^**6**^ **(Mb)**	***P*-value**^**7**^	**SL**^**8**^
5	P1	CSW4	2	104	48.10	1.32-4.46	31.57-41.62	<0.05	*
9	P1	CAEC	46	56	118.85	6.35-8.848	14.00-16.64	<0.0001	***
9	P1	CSW5	45	52	58.07	6.35	11.64-14.01	<0.0001	***
15	P1	CAEC	8	29	112.14	4.23-5.16	7.46-8.85	<0.005	**
15	P1	CSW4	20	33	48.40	6.15-6.94	7.46-8.85	<0.01	*
22	P1	CAEC	0	28	84.75	1.17	1.53-2.63	<0.05	*
22	P1	CSW5	29	42	59.70	2.63	2.63-3.19	<0.0001	***
24	P1	CAEC	23	51	87.78	3.56	5.13-6.13	<0.05	*
24	P1	CSW4	44	51	38.59	5.13	5.13-6.13	<0.0001	***

^1^Chromosome number.

^2^Location of the first QTL.

^3^Location of the second QTL.

^4^Likelihood Ratio Test.

^5^Positions of the two markers flanking the first QTL peak.

^6^Positions of the two markers flanking the second QTL peak.

^7^*P*-values of the H0 vs H2 test obtained by 1000 chromosome-wide permutations.

^8^ H0 vs H2 significance level: *significant at P < 0.05 at the chromosome-wide level, ** significant at P < 0.01 at the chromosome-wide level, *** significant at P < 0.05 at the genome-wide level.

Two-QTLs analyses led to significant QTLs on six chromosomes in P1, specifically chromosomes 5 (CSW4), 9 (CAEC, CSW5), 15 (CAEC, CSW4), 22 (CAEC, CSW5) and 24 (CAEC, CSW4). In P2, no QTL was detected with the two-QTLs analysis.

### Positional candidate genes

Positional candidate genes identified using the software AnnotQTL [13] are listed in Table 4. A high number of genes were identified in each interval. We chose to focus only on genes known to be involved in the immune response, including those controlling signaling pathways. MiRNAs which have the potential to play a role in immune mediation were also included. Genes listed can therefore be considered as both positional and functional candidate genes. No additional evidence for the involvement of these candidate genes in *Salmonella* carrier state or colonization has currently been documented.

**Table 4:** List of genes involved in the immune response identified between the markers flanking the peaks of the QTL detected

**Chicken chr.**^**1**^	**QTL position** **FM**^**2**^**position**	**Human chr.**^**3**^	**Start (pb)**	**Gene ID**^**4**^	**Symbol**	**Description**	**Ensembl ID**^**5**^
2	38.06 Mb/ 86 cM 31.78-38.35 Mb	7	32,053,542	777943	MIR148A	microRNA mir-148a	ENSGALG00000018281
		7	32,326,549	420629	SKAP2	src kinase associated phosphoprotein 2	ENSGALG00000011051
		7	32,584,271	-	gga-mir-1732	microRNA-mir-1732	ENSGALG00000025254
		7	32,586,148	777944	MIR196-2	microRNA mir-196-2	ENSGALG00000018282
		7	32,879,740	395490	TAX1BP1	Tax1 (human T-cell leukemia virus type I) binding protein 1	ENSGALG00000011123
		7	33,469,922	428436	TRIL	TLR4 interactor with leucine-rich repeats	-
		7	33,567,743	428437	CHN2	chimerin (chimaerin) 2, involved in Rac signalling	ENSGALG00000011164
		3	33,985,086	420640	ANKRD28	ankyrin repeat domain 28 (promotes cell migration)	ENSGALG00000011226
		3	34,275,134	395109	RFTN1	raftlin, lipid raft linker 1 (B cell-specific major lipid raft protein)	ENSGALG00000011241
		3	34,473,226	420645	PLCL2	phospholipase C-like 2, involved in MAPK pathway and PI3K signalling	ENSGALG00000011248
		3	35,783,173	420649	RAB5A	RAB5A, member RAS oncogene family, involve in endocytosis	ENSGALG00000011272
		3	37,167,138	420654	NKIRAS1	NFKB inhibitor interacting Ras-like 1	ENSGALG00000011289
19	3.38 Mb/ 21 cM 2.71- 3.80 Mb	1	2,823,176	417489	LAT2/ NTAL_CHICK	Linker for activation of T cells family, member 2	ENSGALG00000001305
		17	3,408,203	395930	RABEP1/ RABAPTIN1	rabaptin, RAB GTPase binding effector protein 1	ENSGALG00000001737
23	0.84 Mb/ 1 cM 0.72- 1.32	1	843,249	428208	HIVEP3	Human immunodeficiency virus type I enhancer binding protein 3	ENSGALG00000000635
26	2.95 Mb/ 29 cM 2.60-3.31 Mb		2,896,047	-	gga-mir-205a	microRNA-mir-205a	ENSGALG00000018312
		1	2,976,923	419862	TRAF3IP3	TRAF3 interacting protein 3, TNF-receptor associated interacting protein	ENSGALG00000001373
		1	2,989,458	419863	IRF6	Interferon regulatory factor 6	ENSGALG00000001405

^1^ Chicken chromosome.

^2^ Flanking markers.

^3^ Human chromosome.

^4^ Gene identification in NCBI database.

^5^ Identification of gene in Ensembl database.

## Discussion

Results of QTL detection are dependent, amongst other parameters, on the statistical method used for analysis. In this study, we used maximum likelihood analyses with the QTLMap software [10][11], whereas regression analysis with the QTLExpress software [8] was used in our previous studies [3,4]. The two approaches were expected to give partially different results. Although we used the same data as previously described [4], genotypes were enriched with eleven SNP markers flanking QTLs for resistance to *Salmonella* colonization.

### Numerous QTLs identified using QTLMap

QTLExpress assumes the F0 lines to be inbred, particularly with the F2 inbred option of QTLExpress used previously [4]. This contrasts to QTLMap which takes into account the alleles segregating within the lines, both between and within sire families. This is of particular importance since the N and 6_1_ lines are highly but not totally inbred, possibly accounting for the reason why more genome-wide QTLs were found in this study than previously observed [4]. Nadaf et al. achieved a similar result with the same two software packages [14]. They analyzed a F2 cross between lines largely divergent for a phenotype of growth, but not fixed for the QTL alleles. They obtained similar results: with QTLMap, they found 5 genome-wide and 4 chromosome-wide significant QTLs, while with QTLExpress there were only 3 and 2 respectively. Two of the QTLs found to be of chromosome-wide significance with QTLExpress were estimated as genome-wide significant with QTLMap. As suggested by the authors, these differences probably originate from the allele segregation within each line. In our case, although segregation was expected to be less frequent since the lines were partly inbred, QTLMap allowed identification of more QTLs with higher significance.

Numerous QTLs were detected with the none vs two QTLs analysis. The latter deals with the possibility that two linked QTL located within a very short interval could not be detected because they are exerting antagonistic effects. Except for CSW4 on GGA5, for the other traits and chromosomes the distance between the two QTLs was rather short and ranged from 7 to 28 cM. Moreover, on several chromosomes, QTLs controlling different traits were observed in close proximity, which supports the reliability of our results. For instance, on chromosome 9, both QTLs controlling CAEC (46 cM; 56 cM) were very close to those involved in the control of CSW5 (45 cM; 52 cM). On microchromosome 22, two pairs of QTLs were identified for CAEC (0 cM; 28 cM) and for CSW5 (29 cM; 42 cM). On chromosome 24, one of the QTLs controlling CAEC and one of the QTLs controlling CSW4 co-localized at 51 cM. All these co-localisations suggest that these regions may indeed have an impact on the control of *Salmonella* carrier-state.

QTLMap software makes it possible to test binary data, such as the presence/absence of bacteria. The distribution of such traits is far from being Gaussian, while normality is assumed for most methods of QTL research. In this study, this question was of special interest for cloacal swabs at 4 and 5 weeks p.i., for which only 41.6 % and 33 % of animals respectively were positive for the presence of bacteria. When analyzing the binary trait CSW4_d_, with the model dedicated to discrete data, only one significant QTL was found on chromosome 23 at 1 cM, close to the QTL detected for CAEC at 10 cM. This QTL, although highly significant at the chromosome-wide level, was not significant at the genome-wide level. Another QTL was detected on chromosome 21 with CSW4_d_ after deleting the three sire families which had the lowest likelihood ratio. It was only significant at the chromosome-wide level, most probably because of a lack of power due to the loss of information when categorizing the data. Since this QTL co-localizes with QTL identified for CSW4 with the one-QTL analysis, it is probably not a statistical artefact.

### Comparison with previous results obtained with QTLExpress

Although a high number of QTLs were identified with each of the software packages, only one QTL on chromosome 22 was identified with both of them. On this chromosome, the QTL for CSW5 identified at 29 cM by the two-QTLs analysis is located at the same position as the QTL found by [4]. We could not identify the dominant QTLs observed by [4] with QTLExpress, since QTLMap does not take dominance effects into account. In particular, for the most significant QTL detected on chromosome 14 for CAEC, dominance effects were very high (d = 0.90) while additive effects were very low (a = −0.11), with a ratio d/2a equal to −4.25. This is probably why it could not be detected by the additive model with QTLMap. Moreover, some of these dominant QTLs could also be false positives as observed by [15]. When analyzing, with a large set of methods, a set of simulated data with only additive QTLs, several dominant QTLs were observed [16]. Conversely, using QTLMap we detected a QTL on chromosome 2 at 86 cM, which was not observed previously [4] using the same set of SNP markers with QTLExpress (except the 3 additional markers close to QTLs observed by Fife *et al*. [7]). However, a QTL for CSW4 was previously detected at 87 cM using microsatellites [2,3]. The lack of detection of this QTL in the previous QTLExpress analysis [4] could be due to the poor information content of several sire families at markers surrounding the QTL region, which is best taken into account by the QTLMap software. The map density and the information content of markers are lower in this region of chromosome 2 than in other regions. For all other QTLs, it must be assumed that either they are statistical artefacts or they display effects so weak that they are significant with only one of the two methods of analysis tested.

Similarly to Calenge *et al.*[4], we identified a very low number of QTL in P2. This may be as a result of the lower number of traits measured (one versus three). However, even when considering only the trait CAEC measured in P2, there were fewer QTLs detected in P2 than in P1. This might be related to the phenotypic distributions, which are highly different in P1 and P2. In P1 most animals were free of *Salmonella* (76.2 %), while in P2 most animals were infected (92.3 %). Despite the fact that, in both P1 and P2, the logarithm of numbers of colony forming units was measured, the biological meaning of both traits differs. The former trait is related to capacity for bacterial clearance: more resistant animals are free from bacteria while the susceptible individuals remain infected. The latter refers to the level of infection: the more resistant animals can maintain the infection at a lower level. Mechanisms underlying both traits may thus be quite different and the same holds for the genes controlling them [16].

### Are *Salmonella* carrier-state and colonization controlled by common loci?

Although most QTLs identified in the present study were not identified in the previous QTLExpress analysis, some of them co-localize with QTLs or candidate genes identified in other, independent studies, which strengthens their interest and reliability.

The QTL on chromosome 3 is of particular interest. Although it was not significant at the genome-wide level, it was the more significant of the two QTLs detected in P2. In addition, it co-localizes with a QTL for resistance to *Salmonella* colonization identified at 96 Mb (vs 94.5 Mb) for the same measure (caecal bacterial count) assessed after infection with another *Salmonella* serotype at an earlier interval post-inoculation (5 days p.i.) [7]. Interestingly, when SNP markers surrounding the QTLs for resistance to colonization were added to the genetic map, the significance of the QTL for resistance to carrier-state reaches the genome-wide significance level. This might be due to the additional information brought by these markers, one of which may be close to the causal gene for the QTL. Nevertheless, two of the three additional SNP markers were in strong segregation distortion, and it therefore cannot be excluded that skewed segregations cause an artificial increase of the QTL significance. Indeed, one of the distorted markers was mapped at an incorrect position when considering physical positions of markers (inversion with an adjacent marker). However, this result is coherent with the co-localisation of this QTL and several candidate genes involved in innate immunity like the avian beta-defensins *AvBD1* to *AvBD14* between 110.20 Mb and 110.27 Mb. Moreover, polymorphisms in four genes within the beta-defensin cluster (*AvBD3, 11, 12* and *13*) were associated with caecal bacterial load in chickens orally infected with *S.* Enteritidis, while *AvBD5* was associated with spleen bacterial load [17,18]. Other genes like the interleukins *IL-17A* and *IL-17 F* are also located in close proximity to the QTL (at 110.36 Mb and 110.37 Mb respectively) and were associated with caecal and spleen bacterial loads, as well as antibody response to *Salmonella* Enteritidis vaccine [17,18]. The role of this immune pathway in resistance to salmonellosis is strengthened by the detection in chromosome 2 of RFTN1, which modulates T cell receptor signals and which is necessary for the fine-tuning of T cell-mediated immune responses and especially the Th17 immune response [19].

Numerous genes related to immunity can be identified between the markers flanking the QTL present on chromosome 2 in P1, which is located adjacent to a QTL associated with the presence of a hardened caseous caecal core after infection with *Salmonella* Typhimurium [7]. Although the QTL peaks are located 10 Mb apart and their confidence intervals do not overlap, QTL locations are known to vary according to many parameters. In this meta-analysis, infection protocols and *Salmonella* serotypes were different, as were the parents of the progenies. Although belonging to the same lines, separation of these flocks may have, over time, resulted in divergence, as strongly suggested by the incomplete segregation of some of the new SNP markers added to our original SNP set. The number of markers used in both of these studies and the strong linkage disequilibrium in these mapping populations may also impact on the resolution of the QTLs. It is therefore possible that both QTLs for these disparate traits do co-localize. Moreover, the gene *IL-6* (interleukin-6), whose product drives induced innate responses in chickens and especially the Th17 pathway [7], is located close to the QTL peak (30.9 Mb with the QTL peak at 38.5 Mb). It is interesting to note that numerous genes identified between the markers flanking the peaks of the QTL present in chromosome 2 are involved in the immune response. Some genes play a role in the Toll-like receptor (TLR) signaling cascade, such as the TLR4 interactor with leucine-rich repeats (TRIL) gene, known to interact with the TLR4 protein. TLR4 is involved in stimulation of immune responses after interaction with bacterial lipopolysaccharide [20],. Moreover, higher TLR4 expression has been observed in caecal cells from an uninfected resistant chicken line compared to those from the susceptible line [16]. Other genes are related to stimulation of this innate immune response pathway, such as TAX1BP1, a key regulator of the NF-κB and IRF3 signaling and involved in anti-apoptotic activities [21]. Interestingly, other genes located on other chromosomes and close to QTL are also involved in these immune signaling cascades. This is the case for TRAF3IP3, which interacts with TRAF3, a regulator of the JUN N-terminal kinase (JNK) and NF-κB which is a transcriptional factor important for interleukin production [22]. Similarly, HIVE3, a member of the ZAS family, which interacts with TRAF1 and TRAF2 to regulate IL-2 expression, was also detected [23].

Surprisingly, numerous genes identified between the markers flanking the peaks of the QTL are involved in cell signaling leading to cell migration and cell proliferation and thus involved in the immune response. This is the case of the miRNA mir148A which promotes cell proliferation and cell cycle progression by targeting p27, a key inhibitor of the cell cycle. The roles of the other miRNAs (mir1732, mir196-2, mir 205a) detected in our study are not known. However, very recent studies in mice suggest that miRNAs are involved in the specific host response to bacterial pathogens such as *Salmonella*.[24]. In a similar way, SKAP2, which is an adaptor protein, plays an important role in cellular functions, such as cell proliferation, migration, and apoptosis [25]. It is important to note that several genes flanking the peaks of our QTL and involved in cell proliferation/migration signaling pathways belong to the Ras GTPases superfamily, also called small GTPases. These small GTPases generally serve as molecular switches for a variety of cellular signaling events but are also modulated by *Salmonella* to drive its entry into cells [26,27]. The Rab family, a subfamily of the Ras GTPases superfamily, regulate many steps of membrane traffic, including vesicle formation and vesicle movement along actin and tubulin networks. These proteins are also crucial for intracellular survival of *Salmonella*[27]. Two genes (RAB5A and RABEP1) corresponding to different QTL, located on different chromosomes, have similar function and belong to the same signaling cascade. Rab5A, indeed, interacts with RABEP1 [28]. Rab5a is a key molecule for the IFN-γ promoted clearance of pathogenic bacteria but it has been also described that the *Salmonella* effector protein SopB promotes phosphatidylinositol 3-phosphate formation on *Salmonella* vacuoles by recruiting Rab5. Within this signaling cascade we could also introduce PLC2 which is a phospholipase C involved in phosphatidylinositol 3-kinase signalling. CHN2 or beta2 chimaerin is a member of the RhoGAP protein family, another Ras GTPase subfamily, showing specific GTPase-activating protein (GAP) activity toward Rac which induces the formation of actin-rich lamellipodia protrusions involved in cell migration [29]; ANKRD28 (a protein that contains twenty-six ankyrin domain repeats) interacts with DOCK180, a guanine-exchange factor of Rac, to promote cell migration [30], and NKIRAS1 is a small GTPase modulating NF-κB activity [31]. Further investigations are needed to elucidate their putative role in the control of resistance to *Salmonella* carrier-state in these populations, with reference to the traits measured in this study.

These co-localisations on chromosomes suggest that these regions may be involved in general mechanisms of resistance, acting on both colonization and carrier-state and effective on both serotypes (Enteritidis and Typhimurium). This result is not surprising: limiting bacteria colonization shortly after infection could be one of the ways to limit bacteria carriage several weeks after infection. Another way of limiting carriage could be, for instance, high bacterial clearance ability. Genes involved in the immune response are interesting candidates for the causative genes underlying these QTLs and warrant further research. Nevertheless, due to the high number of putative candidate genes, a finer mapping of the QTL will be necessary before studying the actual implication of one or several of these genes in resistance to carrier state.

On chromosome 5 the QTL identified at 104 cM for CSW4 in the two-QTL analysis is close to the QTL detected by Tilquin *et al.* at 111 cM for CSW5 [2]. In addition, similarly to both Tilquin *et al.*[2] and Calenge *et al.*[4], we observed a suggestive QTL for CAEC at the same location (108 cM). Nevertheless, these QTL effects are too weak to be considered of interest for further research.

Even with QTLMap, no QTL could be detected on chromosome 7 close to the *SLC11A1* gene (previously named *NRAMP1*), which was shown to be involved in the resistance to both acute salmonellosis [32]‐[34] and *Salmonella* carrier-state [35]. This result is consistent with previous observations [2]‐[4]. It can be assumed that either this gene does not segregate in this cross or its effect is too weak to be detected.

## Conclusions

We observed numerous QTLs for resistance to *Salmonella* carrier-state using maximum likelihood. The role of several genomic regions in the control of *Salmonella* carrier-state was confirmed, consistent with previous findings, while testing the existence of two segregating QTLs allowed the identification of novel regions. The observed differences with QTLExpress were most probably due to the different hypotheses concerning allele segregation, to the strong dominance effect of some of the QTL and to the weakness of some QTL effects. In this context, the most interesting QTLs are probably those that were identified several times in independent studies, even more so when they also co-localize with candidate positional or functional genes. This is why the QTL regions identified on chromosomes 2 and 3 are of particular interest for further research and potentially for marker assisted selection. In addition, their involvement in the resistance to both *S.* Typhimurium colonization and *S*. Enteritidis carrier-state is particularly interesting for selection purposes.

## Competing interest

The authors declare that they have no competing interest.

## Authors’ contributions

ST performed QTL analyses and drafted the manuscript. FC and CB contributed to data analyses and to manuscript drafting. MF, NS and PK contributed to obtaining the marker data. PV performed the *Salmonella* challenges and contributed to the candidate gene analysis. AV obtained the original set of SNP data. ELD contributed to the QTLMap analyses. All authors contributed to the revision of the manuscript and approved the final manuscript.
